# Ototrauma induces sodium channel plasticity in auditory afferent neurons

**DOI:** 10.1016/j.mcn.2011.06.005

**Published:** 2011-09

**Authors:** Alistair G. Fryatt, Mike Mulheran, Julie Egerton, Martin J. Gunthorpe, Blair D. Grubb

**Affiliations:** a Department of Cell Physiology and Pharmacology, University of Leicester, University Road, Leicester, LE1 9HN UK; b Neurosciences Centre of Excellence for Drug Discovery, GlaxoSmithKline Research & Development, New Frontiers Science Park, Harlow, Essex, CM19 5AW UK

**Keywords:** Cochlea, Immunohistochemistry, qPCR, Spiral ganglion neurons, Voltage-gated sodium channels, Noise-induced hearing loss

## Abstract

Exposure to intense sound can cause damage to the delicate sensory and neuronal components of the cochlea leading to hearing loss. Such damage often causes the dendrites of the spiral ganglion neurons (SGN), the neurons that provide the afferent innervation of the hair cells, to swell and degenerate thus damaging the synapse. In models of neuropathic pain, axotomy, another form of afferent nerve damage, is accompanied by altered voltage-gated sodium channel (VGSC) expression, leading to neuronal hyperactivity. In this study, adult Wistar rats were exposed to noise which produced a mild, 20 dB hearing threshold elevation and their VGSC expression was investigated. Quantitative PCR showed decreased Na_V_1.1 and Na_V_1.6 mRNA expression in the SGN following noise exposure (29% and 56% decrease respectively) while Na_V_1.7 mRNA expression increased by approximately 20% when compared to control rats. Immunohistochemistry extended these findings, revealing increased staining for Na_V_1.1 along the SGN dendrites and Na_V_1.7 in the cell bodies after noise. These results provide the first evidence for selective changes in VGSC expression following moderate noise-induced hearing loss and could contribute to elevated hearing thresholds and to the generation of perceptual anomalies commonly associated with cochlear damage, such as tinnitus and hyperacusis.

## Introduction

The Royal National Institute for Deaf People (RNID) estimates that 12.6 million people are affected by noise-induced hearing loss (NIHL) in the UK. Many animal models of NIHL have characterised the effects of intense noise stimuli (≥ 120 dB SPL) presented over several hours or days on the function of the peripheral and central auditory pathways (Duan et al*.*, 2008; Hamernik et al*.*, 1980; Kojima et al*.*, 2007; Salvi et al*.*, 1979; Sullivan and Conolly, 1988). In such studies, profound or complete deafness is elicited and the changes observed in such models are loss of outer and inner hair cells (OHC and IHC, respectively), physical rupturing of cochlear structural membranes (Henderson and Hamernik, 1995; Spoendlin, 1971), and swelling and degeneration of the spiral ganglion neuron (SGN) dendrites resulting from glutamate excitoxicity (Puel, 1995; Puel et al*.*, 1998; Spoendlin, 1971). Damage-evoked neuronal plasticity in the central auditory pathway is also observed with increased spontaneous activity in the dorsal cochlear nucleus (Kaltenbach et al*.*, 2000; Zhang and Kaltenbach, 1998) and inferior colliculus (Salvi et al*.*, 2000).

The pathophysiological consequences of less intense acute noise stimuli (≤ 115 dB SPL), where more modest hearing loss occurs, are much more relevant to human environmental exposure but have not been well characterised. In such models, little physical damage is seen and only modest structural changes in the cochlea occur, including sparse hair cell loss (Hu et al*.*, 2009) and limited SGN degeneration (Kujawa and Liberman, 2009) although further investigation is required to fully understand the effects of such stimuli.

Voltage-gated sodium channel (VGSC) α-subunits (Na_V_1.1–1.9) play an important role in regulating neuronal excitability. Neuronal VGSC α-subunits have been classified as either “central” (Na_V_1.1, 1.2, 1.3, 1.6) or “peripheral” (Na_V_1.7, 1.8, 1.9) subunits, based on their expression patterns. We previously showed Na_V_1.1, Na_V_1.6, Na_V_1.7 subunits are expressed in rat SGNs (Fryatt et al*.*, 2009), a unique VGSC phenotype that conforms to neither the conventional central or peripheral expression patterns.

In peripheral nociceptors, nerve injury and deafferentation induce profound changes in the expression and subcellular distribution of Na_V_1.8, Na_V_ 1.9 (Dib-Hajj et al*.*, 1999; Novakovic et al*.*, 1998; Sleeper et al*.*, 2000) and Na_V_1.3 (Black et al*.*, 1999; Dib-Hajj et al*.*, 1996; Waxman et al*.*, 1994). This strongly influences pain fibre excitability, leading to spontaneous pain, hyperalgesia and allodynia. We hypothesised that acoustic ototrauma, a stimulus known to damage the peripheral SGN processes (Puel, 1995; Puel et al*.*, 1998; Spoendlin, 1971), has the potential to elicit similar axotomy and injury-related changes in SGN ion channel expression. We postulated that altered expression of VGSC subunits could have profound effects on SGN excitability, perhaps affecting hearing threshold and auditory perception. If changes in VGSC expression are seen following moderate NIHL, this could provide additional avenues of investigation for identifying neuronal correlates of hearing loss and perceptual anomalies, such as tinnitus, as well as novel therapeutic pharmacological targets for such conditions.

Using a new model of moderate NIHL, designed to minimise physical damage to the cochlea, we investigated whether the VGSC cohort expressed in normal rat SGNs (Fryatt et al*.*, 2009) alters following ototrauma. We demonstrate that moderate noise exposure results in elevated hearing thresholds and that this is accompanied by marked changes in endogenous VGSC channel expression at the mRNA level and altered immunohistochemical staining for Na_V_1.1 and Na_V_1.7. These changes represent a novel pathology that could, at least in part, contribute to auditory deficits and changes in auditory perceptions including tinnitus.

## Results

### Hearing thresholds before and after moderate noise exposure

**Fig. 1:**
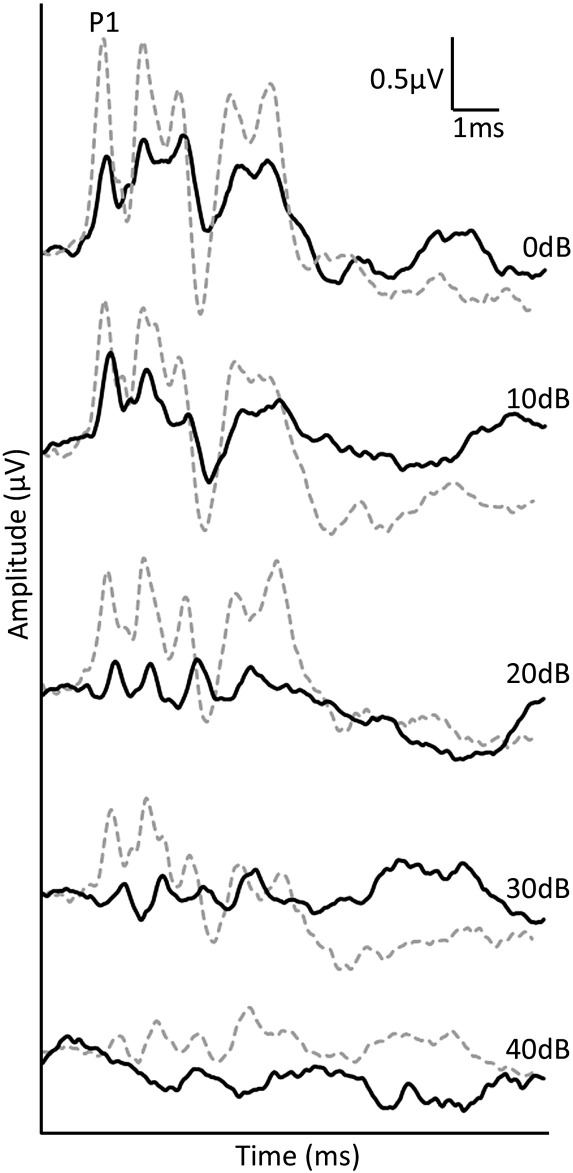
Typical ABR waveforms measured from a rat before and after noise exposure. ABR waveforms in response to 30 kHz tone pips (numbers indicate the attenuation of the tone) before (dashed grey line) and after (filled black line) the complete noise exposure protocol. Following the noise exposure protocol, a decrease in waveform amplitude can be seen at all tone pip attenuations compared to the pre-noise exposure ABR waveforms while no dramatic change in waveform component latencies were observed. Additionally, the ABR waveform could not be reliably identified at 40 dB attenuation following noise exposure.

In order to establish that the noise exposure protocol produced measurable changes in hearing threshold, ABR measurements were taken from all rats used in this study. Representative serial ABR waveforms measured in response to 30 kHz tone pips before and after noise exposure are shown in Fig. 1. The ABR waveform amplitudes decreased dramatically following noise exposure compared to pre-exposure levels. Additionally, the ABR waveform could not be detected at 40 dB attenuation in response to noise exposure, indicating an increase in ABR threshold at this frequency compared to the pre-exposure period.

**Fig. 2:**
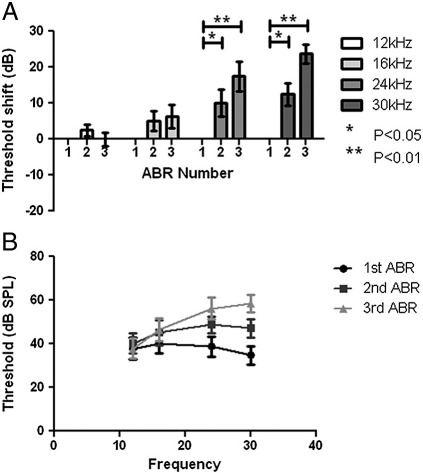
Threshold elevation and hearing thresholds of noise exposed animals. Graphs of mean hearing threshold shifts (A) and hearing thresholds (B) of animals exposed to noise. (A) Hearing threshold for the 24 kHz and 30 kHz tone pips significantly increased during the second and third ABRs, (*, p < 0.05 and **, p < 0.01 respectively) following noise exposure. An increase in hearing threshold for 16 kHz was detected during the third ABR, after two noise exposures (p = 0.09), while the hearing threshold for 12 kHz remained unaffected. Please note that ABR number refers to each trial as described in Fig. 9.

The mean ABR thresholds and threshold elevations following noise exposure (n = 8) are shown in Fig. 2. On completion of the noise exposure protocol, there was no detectable increase in the average hearing threshold at 12 kHz. At 16 kHz, a small increase in ABR threshold of 6 ± 3 dB (p = 0.09) was seen following the complete noise exposure protocol. Marked elevations in ABR threshold were observed at 24 kHz and 30 kHz following both first and second noise exposures. At 24 kHz, the magnitude of the mean threshold elevations was 10 ± 4 dB (p < 0.05) and 18 ± 4 dB (p < 0.005) after the first and second noise exposures respectively, while the mean elevations for 30 kHz were 13 ± 3 dB (p < 0.005) and 24 ± 3 dB (p < 0.001) respectively.

### Changes in P1 amplitudes following noise exposure

**Fig. 3:**
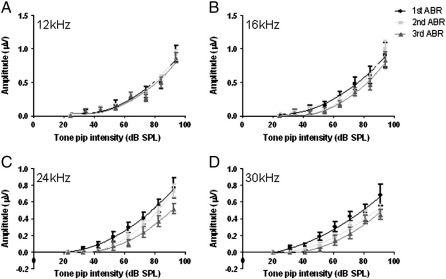
Amplitude of the P1 waveform following tone pip attenuation in noise exposed rats. The mean amplitude of P1 in response to (A) 12 kHz, (B) 16 kHz, (C) 24 kHz and (D) 30 kHz tone pips for each set of ABR measurements and fitted with lines of best fit. For each frequency tested, the amplitude of P1 decreases as the tone pips are attenuated. There was no significant difference between the P1 amplitudes from the ABR measurements at any stimulus intensity in response to the 12 kHz and 16 kHz tone pips. However, significant decreases in P1 amplitude in comparison to the 1st ABR were observed during the second and final ABR experiments in response to the 24 kHz and 30 kHz tone pips (p < 0.05). The fitted lines show no change in the gradient of the curve following noise exposure.

Fig. 3 shows the average P1 amplitudes from the same noise exposed animals in response to discrete tone pip stimulus steps. The P1 amplitudes in response to the 12 kHz and 16 kHz tone pips were not significantly different following the complete noise exposure protocol (p > 0.05). However, testing the 24 kHz and 30 kHz hearing ranges of these animals revealed a significant decrease in P1 amplitude of approximately 35% following the final ABR when compared to the first ABR measurements, especially at the loudest tone pip level used (p < 0.05-0.01).

Using the discrete stimulus attenuation steps, a simple input/output transfer function was fitted using a line of best fit for each frequency. Following the first ABR, a second order polynomial line of best fit was fitted, with the form:

$$y=lx^{2}–mx+c$$

The parameters *l* and *m* in terms of *x* reflected the primary transfer function of the cochlea at the level of the ABR signal while the *c* term is related to sensitivity of the cochlea. Following the second and final ABR measurement, lines of best fit were determined using the same equation as they yielded R^2^ values greater than 0.9. Direct inspection showed no dramatic changes in the gradients of these fitted lines at all frequencies. However, at 24 kHz and 30 kHz, rightward shifts in the fitted curves were identified, consistent with the significant elevations in ABR threshold. The simplest interpretation of the lines of best fit is that the cochlea exhibits some modest damage but is still functional.

### P1 latency following noise exposure

**Fig. 4:**
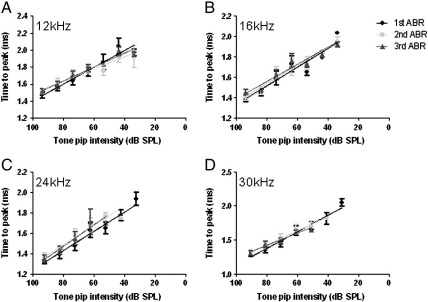
Latency of the P1 waveform following tone pip attenuation in noise exposed rats. The mean latency of P1 in response to (A) 12 kHz, (B) 16 kHz, (C) 24 kHz and (D) 30 kHz tone pips for each set of ABR measurements and fitted with lines of best fit. For each frequency tested, the time to the peak for P1 increases as the tone pips are attenuated. There was no significant difference between the P1 latency from the repeated ABR measurements in response to the tone pips across all frequencies (p > 0.05). The fitted lines show no change in gradient following noise exposure.

In addition to P1 amplitude, P1 latency was also measured at each tone pip intensity level. There was no significant change in the average P1 latency with tone pip attenuation, as shown in Fig. 4. As before, lines of best fit were determined for the average P1 latencies for each frequency following the first ABR measurements, using a line of best fit with the form:

y=mx+c

The parameter *m* with respect to *x,* reflects the coefficient of the travel and processing time of the P1 wave and *c* is a relative measure of the absolute refractory period of the cochlear nerve, neuronal recruitment and synchrony as well as acoustic and travelling wave delays. Following the second and final ABR measurement, lines of best fit were determined using the same equation as they again yielded R^2^ values greater than 0.9. Direct inspection showed no dramatic changes in the gradients of these fitted lines at all frequencies, indicating that the ototrauma had not damaged the ability of the cochlea to transduce sound stimuli, although the threshold elevations clearly demonstrate that the sensitivity of the cochlea to sound stimuli was reduced.

### Gene expression in spiral ganglion neurons

Primers and probes for three housekeeping genes were designed and used. Robust signals for β-actin, cyclophilin A and GAPDH were detected from the modioli cDNA isolated from control (n = 6) and noise exposed (n = 7) rats (see supplementary material), with no significant difference detected in the housekeeping gene expression between the two groups. From these results, β-actin was used as the reference gene for the calculation of fold changes in VGSC mRNA in the test samples.

### Voltage-gated Na^+^ channel mRNA expression following noise exposure

**Fig. 5:**
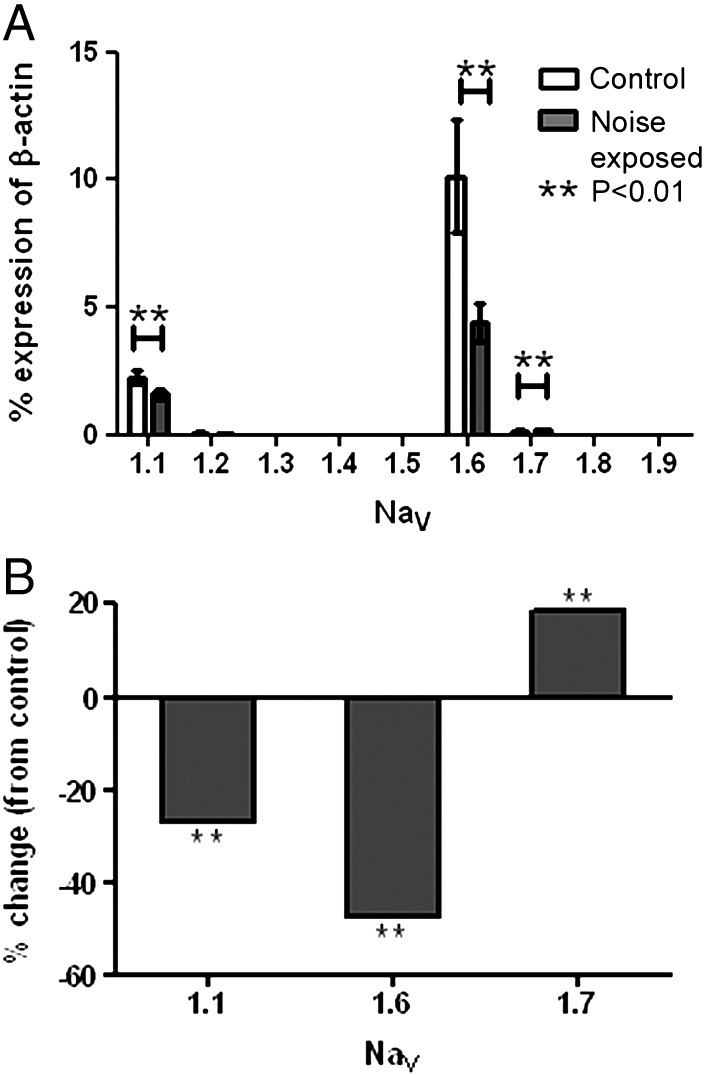
Quantitative PCR results showing fold change of voltage-gated Na^+^ channel expression following noise exposure. (A) Graph showing voltage-gated Na^+^ channel mRNA expression from the modioli of control (clear bars, n = 6) noise exposed (filled bars, n = 7) animals shown as a percentage of β-actin expression. The expression level of Na_V_1.1 and Na_V_1.6 both significantly decreased (**, p < 0.01) following noise exposure while Na_V_1.7 RNA expression increased following noise exposure (**, p < 0.01) and mRNA for the other isoforms was not detected (n = 7). (B) Graph detailing the fold change of Na_V_1.1, Na_V_1.6 and Na_V_1.7 mRNA expression in the noise exposed animals. Each sample contained the mRNA isolated from a pair of modioli collected from one animal.

Fig. 5 *shows* that PCR products for Na_V_1.1, Na_V_1.6 and Na_V_1.7 were detected in the modioli of control and noise-exposed rats, confirming previous RT-PCR experiments that demonstrated the presence of the same isoforms (Fryatt et al., 2009). Fig. 5 also shows a statistically significant reduction in gene expression of 29% for Na_V_1.1 (p < 0.001) and of 56% for Na_V_1.6 (p < 0.001) compared to the control modioli following the noise exposure protocol. Additionally, there was a modest but significant increase in Na_V_1.7 gene expression following noise exposure of 20% (p < 0.005). Primers and TaqMan probes specific for additional VGSC isoforms did not reveal *de novo* expression of VGSC mRNA in noise exposed cochleae (Fig. 5A) and RT-PCR experiments as conducted previously (Fryatt et al*.*, 2009) support this observation (data not shown).

### *S*ubcellular localisation of VGSC α-subunits in noise-damaged cochleae

**Fig. 6:**
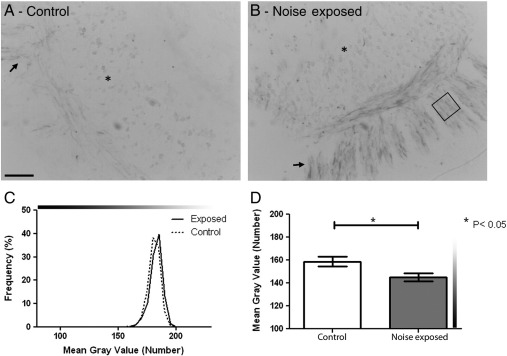
Na_V_1.1 staining in control and noise exposed cochlea. Sections of cochlea from control (A) and noise exposed (B) rats incubated with antibodies against Na_V_1.1. (A) Faint staining of the SGN cell bodies (*) and peripheral processes (arrow) was observed in the control cochlea. (B) Following the noise exposure protocol no change in staining intensity was detected in the SGN cell bodies, although darker staining was seen in the peripheral processes compared to the control cochlea (insets). (C) Graph of SGN cell body staining intensity, showing no difference in the frequency of the mean grey values between the noise exposed and control (dotted line) rats. (D) Graph of the mean peripheral process staining intensity from control (clear bar) and noise exposed (filled bar) rats. Regions of the peripheral processes (box in B) were analysed and their mean grey value measured. This analysis showed a significant increase in the intensity of labelling of Na_V_1.1 in the peripheral processes of noise exposed rats compared to control animals (*, p < 0.05; n = 3 animals in each group). Scale bar = 50 μm, 15 μm in insets. Grey values, 0 = black, 255 = white, indicated by graduated bars.

Using a highly selective Na_V_1.1 antibody, sections of control cochleae showed a similar pattern of staining to that observed previously (Fryatt et al*.*, 2009). Very faint staining of the SGN cell bodies and peripheral processes was observed with more intense labelling of the central processes in cochlear sections from control rats (Fig. 6A).

The sections of cochleae from noise exposed animals showed a different pattern of Na_V_1.1 expression. On visual inspection, staining was still observed in the central processes but much more intense staining was also observed in the peripheral processes (Fig. 6B). There was no detectable change in Na_V_1.1 SGN cell body staining intensity between the noise exposed and control cochleae (Fig. 6C). Morphometric analysis of the noise exposed cochleae confirmed the visual observations with significantly more intense labelling for Na_V_1.1 in the peripheral processes than in similar regions of control cochleae (Fig. 6D). The average mean grey value for the peripheral processes from the noise exposed animals was significantly higher (145.1 ± 3.63) than the control animals (158.7 ± 4.28; p < 0.05).

### Na_V_1.6

**Fig. 7:**
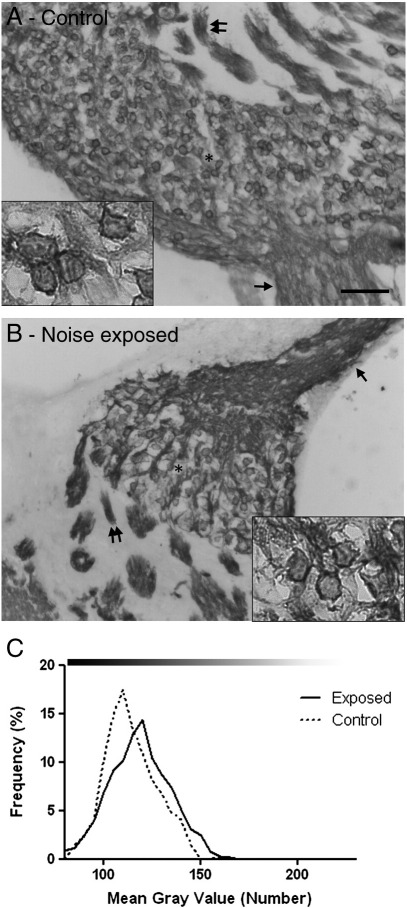
Na_V_1.6 staining in control and noise exposed cochleae. Micrographs of cochlea sections stained for Na_V_1.6 from control (A) and noise exposed (B) animals. Robust staining can be seen in the SGN cell bodies (* and inset), peripheral processes (single arrow) and central processes (double arrow) from the control animals (A). Following noise exposure, cell body, peripheral process and central process staining appears similar to the control animals (B). Frequency pictographs of the SGN cell body mean grey value (C) show no difference in the staining intensity distribution for Na_V_1.6 between the control (dotted line) and exposed (solid line) animals. Scale bar = 50 μm, 15 μm in insets. Grey values, 0 = black, 255 = white, indicated by bar above pictographs.

The staining seen with the Na_V_1.6 antibody in sections of control cochleae was similar to that reported previously (Fryatt et al*.*, 2009). There was very intense labelling of both the SGN central and peripheral processes as well as the cell bodies (Fig. 7A). On visual inspection of sections of cochleae from noise exposed animals, the pattern of SGN staining was similar to that seen in the unexposed control animals (Fig. 7B), with intense Na_V_1.6 staining in SGN processes and cell bodies. The analysis of cell body staining intensity showed that there was no difference in the intensity of Na_V_1.6 staining between the control and noise exposed SGN (Fig. 7C). Using light microscopic techniques, it was not possible to determine changes in Na_v_1.6 staining levels at nodes of Ranvier in the auditory nerve and we cannot rule out the possibility that protein expression was altered at this location.

### Na_V_1.7

**Fig. 8:**
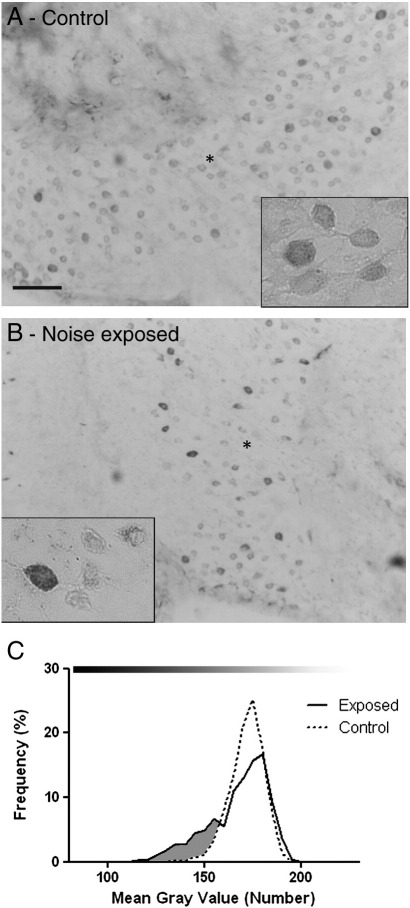
Na_V_1.7 staining in control and noise exposed cochleae. Micrographs of cochlea sections stained for Na_V_1.7 from control (A) and noise exposed (B) animals. Robust staining can be seen in the SGN cell bodies (* and inset) from the control animals (A). Frequency pictographs of the SGN cell body mean grey value (C) show an increase in frequency of the darker stained neurons (shaded region) following noise exposure (solid line) compared to the control SGN (dotted line). Scale bar = 50 μm, 15 μm in insets. Grey values, 0 = black, 255 = white, indicated by bar above pictographs.

The Na_V_1.7 staining of SGN from control cochleae was similar to that reported previously (Fryatt et al*.*, 2009), with robust labelling observed in the cell bodies (Fig. 8A). Following the noise exposure protocol, no increase in staining for Na_V_1.7 in the central or peripheral processes was observed (Fig. 8B). However, at the level of the SGN cell bodies 50% of the noise exposed cochleae analysed appeared to have a number of darker labelled SGN compared to control cochleae (3 out of 6 animals), an observation confirmed by subsequent morphometric analysis (example in Fig. 8C). Of the remaining animals analysed, one showed no change in cell body staining, one showed an increase in frequency of lighter stained neurons and the last showed an increase in the frequency of darker and lighter stained SGN, compared to the control cochleae.

In addition to the morphometric analysis conducted above, cell counts were performed to identify any change in SGN cell body number between the control and noise exposed cochleae. There was no significant difference in the number of SGN cell bodies in the noise exposed cochleae when compared to the control, unexposed cochleae (data not shown).

### Control results

Immunohistochemistry antibody validation is vital for accurate interpretation of the experimental results. As conducted previously (Fryatt et al*.*, 2009), sections of cochleae incubated either without the primary antibody or with the primary antibody preincubated with the respective control peptide did not reveal any non-specific staining attributable to the secondary antibodies. Previous analysis of the tissue distribution of VGSC isoforms in the mammalian nervous system (Catterall et al*.*, 2005) identified positive control tissues to validate the VGSC antibody binding. The antibodies used in this study produced an identical pattern of labelling with Na_V_1.1 staining strongly observed in the Purkinje and granule cell layers of rat cerebellum (Whitaker et al*.*, 2001) and Na_V_1.6 staining in the Purkinje cell soma of the cerebellum (Schaller and Caldwell, 2000) with membranous labelling of large diameter dorsal root ganglion (DRG) neurons (Tzoumaka et al*.*, 2000). Na_V_1.7 staining was observed, as previously reported (Coward et al*.*, 2001; Djouhri et al*.*, 2003), in small, medium and large diameter DRG neurons (data not shown).

## Discussion

### Altered voltage-gated Na^+^ channel expression accompanies moderate ABR threshold elevation

The results presented here provide the first demonstration that the expression of VGSCs can be altered in rat SGN following acute acoustic trauma that generates a moderate hearing loss. This elevation in ABR threshold seen here can be largely attributed to damage at the level of the OHCs (Chen and Fechter, 2003; Henderson and Hamernik, 1995). However, the accompanying changes seen in VGSC expression provide preliminary evidence of additional site that may contribute to auditory dysfunction. Any change in VGSC subunit expression at action potential initiation sites or nodes of Ranvier has the potential to influence afferent fibre excitability, thereby modulating P1 wave amplitude. This could conceivably alter neuronal excitability, perhaps resulting in a hypoactive phenotype and reduction in spontaneous neuronal activity, consistent with the reported decrease in spontaneous activity in the cochlea following NIHL (Chen et al*.*, 1996; Liberman and Dodds, 1984).

Such changes have the potential to contribute to hearing loss and perceptual anomalies such as tinnitus. While tinnitus is considered a symptom of hyperactivity of the central auditory pathway (Bauer et al*.*, 2008; Kaltenbach et al*.*, 2004; Turner et al*.*, 2006; Zhang and Kaltenbach, 1998) through plastic changes arising from the loss of excitatory SGN signals, increased spontaneous activity in the inferior colliculus neurons following noise exposure has been shown to only occur with the presence of a functional cochlea (Mulders and Robertson, 2009), indicating a role for the cochlea in tinnitus generation following NIHL. Additionally, any change in VGSC expression in the SGN could profoundly alter the excitability of these neurons, perhaps resulting in a hypoactive phenotype and reduction in spontaneous neuronal activity, consistent with the reported decrease in spontaneous activity in the auditory nerve following NIHL.

These results also show that auditory afferent neuronal response to ototrauma has parallels with the somatosensory afferent response to mechanical damage with changes in VGSC expression occurring in both. However, the changes in SGN VGSC following ototrauma are concerned with transient Na^+^ current generation, whilst those resulting from damage to somatosensory afferents affect persistent Na^+^ currents, indicating that subtle differences exist between these two systems.

Future experimentation employing the current noise model would require *in vivo* and *in vitro* electrophysiological recording to determine how the any changes in Na_V_1.1, Na_V_1.6 and Na_V_1.7 expression seen here affect excitability SGN.

### Moderate noise exposure affects cochlear sensitivity without severely damaging the cochlea

The noise exposure protocol generated peak threshold elevations in the ABR of about 20 dB at higher frequencies, although suprathreshold gradients of P1 amplitude and response latencies remained comparable with control values. This supports the interpretation of a primary effect of the noise exposure as a moderate loss in cochlear OHC sensitivity, but with considerable retention of cochlear function. This decrease in sensitivity most likely reflects disruption and derangement of the OHC stereocilia and some degree of OHC loss (Chen and Fechter, 2003; Henderson and Hamernik, 1995; Hu et al*.*, 2000). With reference to human hearing, the measured deficit of approximately 20 dB on average at the higher frequencies tested would be considered a mild loss of hearing sensitivity (Niskar et al*.*, 1998). Although the hearing thresholds were elevated at the end point of this model, additional ABR measurements after longer recovery periods are needed to establish whether these elevations are permanent.

The noise exposure protocol developed in this study can be considered to be less traumatic when compared to other NIHL studies. The use of noise exposures at higher sound pressure levels, 120–140 dB SPL, results in a greater degree of deafness than documented here (Heffner and Harrington, 2002; Kojima et al*.*, 2007; Spoendlin and Brun, 1973) by near complete destruction of the reticular lamina and organ of Corti. Thus the use of 110 dB SPL acute noise in the present study would generate a more moderate level of deafness that is analogous to that observed in the human population. Additionally, Spoendlin and Brun (1973) observed severe damage to the organ of Corti of guinea pigs with noise exposures over 130 dB SPL, but reduced hair cell loss using exposure intensities below 120 dB SPL. Interestingly, 115 dB SPL broadband noise for 2 h induced hearing loss in rat cochleae but not the immediate destruction of large numbers of hair cells or supporting cells (Hu et al*.*, 2009). These authors also reported a hearing threshold elevation of 24 ± 5.2 dB at 7 days post-exposure, which is similar to the hearing threshold elevations reported in this study and verifies the protocol used here.

While many studies have investigated the effect of noise exposure above 110 dB SPL on the cochlea, a recent publication by Kujawa and Liberman (2009) has further highlighted some important consequences of a more modest ototrauma protocol. In their study mice were exposed to an octave band noise stimulus (8–16 kHz) at 100 dB SPL for 2 h, followed by physiological testing of hearing function, counting of SGN afferent synapses and cell body numbers. Their results showed a temporary threshold shift with a recovery of hair cell function within two weeks, indicating preservation of the IHC and OHCs. However, there was a decrease in afferent SGN synaptic terminals and a decrease in SGN number over time (several weeks to months) without a decline in hair cell number, which could contribute to decreased firing in the afferent fibres following NIHL. This indicates that exposure to modest level ototrauma can result in accelerated loss of SGN, although not within the timescale of the experiments reported here.

The findings presented by Kujawa and Liberman (2009) in conjunction with the results presented here add weight to the need for more detailed analysis of the consequences of exposure to modest levels of noise in terms of the role and function of the auditory nerve. A more unified, systematic approach must be adopted to investigate the neuronal consequences of exposure to noise that does not result in catastrophic damage to the cochlea since this better reflects the type of noise exposure and ototrauma experienced by people with early hearing loss, *i.e.* elevated hearing thresholds at higher frequencies.

Having established that the noise exposure protocol used in these experiments produced a reduction in cochlear sensitivity over the mid (16 kHz) to high (30 kHz) frequency range without complete disruption of the cochlea, it was interesting to note that VGSC subunit expression was markedly altered in the noise-exposed rats.

### Na_V_1.1 subunit expres*sion*

Na_V_1.1 expression in normal cochleae was predominantly observed in the SGN axons, indicating a possible role in action potential initiation and propagation. Indeed, Na_V_1.1 staining has been shown at axon initial segments as well as co-localisation with Na_V_1.6 at nodes of Ranvier (Duflocq et al*.*, 2008). Following noise exposure, the qPCR results showed a decrease in Na_V_1.1 mRNA of 29% and a marked increase in Na_V_1.1 staining was observed in the SGN peripheral processes, which innervate the hair cells within the organ of Corti. In some respects, this observation parallels the altered expression of Na_v_1.3 (Black et al., 1999) and Na_V_1.8 in sciatic nerve injury, where an increase in VGSC α-subunit staining is seen at the site of neuronal damage through translocation of each protein, events that are believed to underlie the increase in neuronal excitability observed in the damaged nociceptive neurons. Our results suggest that a similar translocation of Na_v_1.1 protein occurred in the SGN following NIHL, which would explain the increase in Na_V_1.1 staining without an increase in mRNA expression, with Na_V_1.1 depleted from cellular stores. However, further investigation is required to establish whether the increase in Na_V_1.1 staining observed following noise exposure results in altered SGN excitability.

### Na_V_1.6 subunit expression

Na_V_1.6 expression in control SGN was observed along the neuronal processes and at the cell body. Following noise exposure, the qPCR results showed a decrease in Na_V_1.6 mRNA of approximately 50%, although there was no corresponding change in immunostaining seen at the cell bodies where staining was very intense. However, this does not necessarily indicate that more subtle changes in Na_v_1.6 localisation have not occurred. For example, it is not practicable to analyse Na_v_1.6 levels at the nodes of Ranvier in the auditory nerve using light microscopy due to their small size. Future experiments incorporating quantitative immunogold labelling of auditory nerve axons combined with electron microscopy will be required to assess changes in Na_v_1.6 expression at the nodes of Ranvier.

### Na_V_1.7 subunit expression

In sections of control cochleae, Na_V_1.7 staining was observed predominantly at the SGN cell body. The qPCR results indicated an increase in Na_V_1.7 mRNA of approximately 20% following noise exposure, which was reflected in the immunohistochemistry results where 50% of analysed cochleae showed an increase in frequency of more intensely labelled SGN cell bodies. This suggests that Na_V_1.7 expression is up-regulated in the SGN following moderate noise exposure. In nociceptive neurons, Na_V_1.7 is believed to regulate the excitability of pain fibres. Mutations in Na_V_1.7 lead to altered pain perception, with gain of function mutations producing hyperexcitable nociceptive neurons and states such as paroxysmal extreme pain disorder (Fertleman et al*.*, 2006) and erythermalgia (Dib-Hajj et al*.*, 2005; Yang et al*.*, 2004), while loss of function mutations produce no sodium current and generate congenital insensitivity to pain (Cox et al*.*, 2006). It is interesting to note that in these studies of human Na_V_1.7 mutations, the patients are reported to have no apparent deficits in autonomic or sensory function, including hearing, although anosmia has been noted in patients insensitive to pain (Hirsch et al*.*, 1995; Nilsen et al*.*, 2009). It is unclear at present if these findings mean Na_V_1.7 is either not expressed in human SGN, is compensated by another VGSC or indeed if the investigators measured the hearing function of the affected individuals in detail and missed subtle abnormalities. Functionally, Na_V_1.7 recovers slowly from fast inactivation and displays slow closed-state inactivation, allowing current flow in response to small, slow depolarisations (Cummins et al*.*, 1998; Herzog et al*.*, 2003), suggesting this subunit may amplify generator potentials. An increase in Na_V_1.7 expression could result in an increase in action potential generation in the SGN, leading to hyperactivity, although further investigation is required.

### Expression of other voltage-gated VGSC isoforms

Animal models of pain have shown that additional VGSCs can be re-expressed following nerve damage, such as Na_V_1.3 (Black et al*.*, 1999; Hains et al*.*, 2003; Hains et al*.*, 2004). During the qPCR experiments, primers and TaqMan probes specific for additional VGSC isoforms were used but did not reveal the *de novo* expression of VGSC mRNA in noise exposed cochleae. Additionally, RT-PCR experiments as conducted previously (Fryatt et al*.*, 2009) were performed but also did not identify the presence of additional VGSC isoforms. Previous studies have shown the expression of Na_V_1.6 in the SGN and Na_V_1.2 in the efferent processes of mouse cochleae (Hossain et al*.*, 2005). In agreement with our previous study (Fryatt et al*.*, 2009), the present experiments have identified Na_V_1.6 and two additional VGSC isoforms expressed in rat SGN. The apparent differences in VGSC cohort expression could simply be due interspecies differences between rats and mice, but it is unclear if VGSC isoforms other than Na_V_1.2 and Na_V_1.6 were examined in the previous mouse investigation.

### Summary

This study provides, for the first time, compelling evidence for plastic changes in VGSC expression in the rat SGN following acute, moderate noise exposure. These changes in VGSC expression may contribute to mild hearing loss and/or tinnitus, as changes of this sort have been shown previously to influence the excitability and firing properties of afferent pain fibres following deafferentation. While this study has focused on VGSC expression, changes in K^+^ channel expression have also been identified in DRG neurons following axotomy injury (Ishikawa et al*.*, 1999). These channels also determine the neuronal electrical properties and investigation of the already established K^+^ channel isoforms in the SGN (Adamson et al*.*, 2002; Chen and Davis, 2006; Davis, 2003) using our noise exposure model would certainly be warranted.

This study has also shown altered VGSC expression six days after noise exposure and certainly warrants further studies to investigate VGSC expression at both shorter (hours/days) and longer (weeks/months) time points than used here to fully characterise any additional changes or recovery of VGSC expression. While no direct measures of SGN excitability were performed in this investigation, future studies will also examine the link between changes in VGSC expression in the SGN, auditory nerve excitability, deafness and abnormal auditory sensations, such as tinnitus, and test potential therapeutic agents, such as VGSC modulators *e.g.* lamotrigine, to prevent or reverse these conditions.

## Experimental methods

### Animal procurement and care

Adult male Wistar rats (150–250 g) were purchased from Charles River UK (Charles River UK Ltd, Margate, UK). Animals were allowed a seven day acclimatisation period before experimental use. Animals were kept in a 12 h light/dark cycle, with minimum natural light, and were given water and food *ad libitum*.

### Pre-anaesthetic preparation

Prior to the administration of the anaesthetic, 40 μg.kg^−1^ atropine (atropine sulphate, Antigen Pharmaceuticals, Croyden, UK) was administered subcutaneously. Ocular lubricating ointment (Lacri-lube, Allergan Ltd, Marlow, UK) was applied to the eyes. The rat was also supplemented with normal saline, given subcutaneously at hourly intervals. Rat body temperature was measured using a rectal probe thermometer (K-type ATK-1319 thermometer with stainless steel probe, ATP Instrumentation Ltd, Ashby-de-la-Zouch, UK) and was maintained between 35.9 and 37.5 °C using a heated pad.

### Anaesthetic administration

Rats were anaesthetised, using neuroleptanalgesia, with Hypnorm (VetaPharma Ltd, Leeds, UK; 0.08 mg·kg^−1^ fentanyl and 2.5 mg·kg^−1^ fluanisone) and midazolam (Hypnovel, Roche Products Ltd, Welwyn Garden City, UK; 1.25 mg·kg^−1^) in a single intraperitoneal (IP) injection. The depth of anaesthesia was tested by monitoring the pedal withdrawal reflex. Evoked auditory brainstem response (ABR) and noise exposure experiments would only begin if the pedal withdrawal reflex was absent, indicating that the animal was deeply anaesthetised. Supplementary top-up injections were given as required.

### Evoked auditory brainstem response measurements

ABR measurements were performed to determine the hearing threshold of each rat. This was achieved using 3 needle electrodes inserted subdermally along the mastoid bone below the left ear (recording electrode), between the ears (reference) and lastly, in the abdomen (earth) in an anaesthetised animal. Tone pips (5 ms duration, frequency range 12–30 kHz) were delivered using an ultra-high performance reverse driven microphone (model 4192, Brüel and Kjær Sound and Vibration Measurement A/S, Nærum, Denmark). The microphone was positioned approximately 5 mm above the entrance to the auditory canal so that the tone pips were delivered directly into the rat′s left ear. The evoked potentials were amplified, filtered and averaged (300 samples). The stimulus was attenuated from the maximal output of the driver in 10 dB steps to 80 dB attenuation (approximately 10 dB SPL) using a digital attenuator (Tucker Davies Technologies, USA), with each step repeated at least twice.

Closed field calibration of the driver was in accordance with manufacturer′s specification. The maximum output of the reverse driven microphone was 94 dB SPL for 12 kHz and 16 kHz, 92.5 dB SPL for 24 kHz, and 91 dB SPL for 30 kHz. These values were referenced to as 0 dB attenuation.

The ABR data were entered into Microsoft Excel® spreadsheets and an averaged waveform was generated for each attenuation step, allowing accurate measurement of the hearing threshold. The hearing threshold level was determined when the components of the ABR waveform could not be detected visually. The amplitude and latency of the waveform component P1, thought to be generated by the cochlea and cochlear nerve activity (Church and Gritzke, 1987; Melcher et al*.*, 1996; Popelar et al*.*, 2008; Starr, 1976), was measured using custom software.

### Noise exposure protocol

Our preliminary studies exploring the effect of up to four acute noise exposures on hearing threshold and VGSC expression showed that two exposures resulted in significant changes in both at six days post exposure. Based on this, and within the constraints of the study, it was decided to focus on developing a two acute exposure model accompanied by measurement of the changes in VGSC expression.

**Fig. 9:**
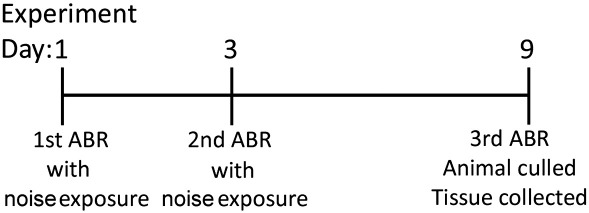
Diagram of the ABR and noise exposure protocol. All noise exposed rats were subjected to the complete protocol above. Control rats were subjected to the same protocol but without the noise exposure sessions.

The anaesthetised rats were placed in a soundproof chamber for exposure to the auditory insult. The auditory stimulus consisted of a single tone (14.8 kHz) applied at 110 dB SPL for 2 h, repeated twice with a 48 h interval in between. The noise exposed rats were sacrificed 6 days following the second noise exposure session, at which point their cochleae were harvested. ABR measurements were taken before each noise exposure session and prior to sacrifice. Control animals were subjected to the same ABR and anaesthesia regimen but were not exposed to the noise stimulus. The complete experimental protocol is described in Fig. 9.

### Quantitative PCR

Noise exposed (n = 7) and control (n = 6) rats were sacrificed using Euthanal® (IP) in accordance with UK Home Office Animals Act 1986 (Schedule 1). The temporal bones were removed and excess bone was removed to leave the bony inner ear segment that contained the cochlea. The otic capsule of the cochlea was removed to reveal the modiolus, which was then carefully removed from the remaining bone and frozen over dry ice in a sterile RNase/DNase free tube.

### mRNA isolation and cDNA synthesis

The two modioli from each animal were pooled for each experiment. The total RNA was isolated using the QIAGEN RNeasy lipid tissue mini-prep kit (QIAGEN, Crawley, UK), as per the manufacturer′s instructions using a QIAGEN tissue lyser to homogenise the samples. A DNase solution was added to digest any remaining DNA (Qiagen RNase-free DNase set) before the mRNA was eluted in RNase free water (Applied Biosystems, Warrington, UK). A NanoDrop ND1000 spectrophotometer (NanoDrop, Wilmingtion, USA) was used to quantify the amount of total mRNA eluted. Total mRNA was transcribed to cDNA using the Applied Biosystems high-capacity cDNA reverse transcription kit using a 96 well plate thermal cycler. The cDNA contained in the well plates was kept at − 20 °C until use. mRNA from each animal was also processed without reverse transcription to identify any genomic contamination of the samples during the PCR reaction.

### PCR reaction

The PCR reaction was performed using the Applied Biosystems 7500 fast thermal cycler Absolute Quantification (AQ) protocol. Rat genomic standards with known copy number (Applied Biosystems), no template control (NTC) and 5 μl of the sample cDNA with (+) and without (−) reverse transcription were pipetted into the appropriate well of a 96 well plate. The Taqman buffer (Applied Biosystems), 10 μM custom designed oligonucleotide primers and Taqman probes (Sigma Genosys, Cambridge, UK) were added to each well, followed by the Taqman master mix (Applied Biosystems). The plates were sealed with an optical adhesive cover, briefly centrifuged and placed in the 7500 fast thermal cycler. The AQ protocol was run and the copy number of the unknown target sequence was calculated by using the standard curve generated from the genomic standards, with the results exported to a Microsoft Excel® spreadsheet.

### qPCR primers

The details of the primer pairs and Taqman probes specific to the VGSC α-subunits and the housekeeping genes used for qPCR are detailed in supplementary material Table 1. The housekeeping genes selected for this study were β-actin, glyceraldehyde-3-phosphate dehydrogenase (GAPDH) and cyclophilin A (also known as peptidylprolyl isomerase A). The fold change in VGSC expression following noise exposure was calculated using the 2^-ΔΔCt^ method (Livak and Schmittgen, 2001), using β-actin as the control gene.

### Cochlear preparation and sectioning

Noise exposed (n = 6) and control (n = 6) male Wistar rats were sacrificed using Euthanal® (IP) in accordance with UK Home Office Animals Act 1986 (schedule 1). Where possible, control and noise exposed rats were processed in parallel so that the cochlear tissues from both animals could be directly compared following immunohistochemical staining. The temporal bones were dissected and excess bone was removed to leave the bony inner ear segment that contained the cochlea. The cochleae were placed in 2% paraformaldehyde solution for 30 min and demineralised in 8% EDTA until soft (4 to 10 days at 4 °C). The cochleae were then placed in PBS with 30% sucrose overnight at 4 °C and then were infiltrated with Tissue Tek OCT overnight at 4 °C (Cho et al*.*, 2004). The fixed cochleae were quickly frozen in Tissue Tek over dry ice/hexane and stored at − 20 °C. Frozen 15 μm sections were cut from tissue blocks, with sections cut at approximately 90^o^ to the modiolar axis, mounted on Polysine™ slides and air dried. Tissue sections were washed (1 × 15 min) in PBS. A whole cochlea was sectioned for each experiment to ensure representative sections from the apex, middle and base. For SGN cell counting purposes, each alternate section was discarded to ensure the same neuron was not counted twice.

### Antibody information

Information about the primary and secondary antibodies used in this study is presented in supplementary material Table 2.

### DAB/HRP immunohistochemistry

Detection of VGSC subtypes was performed as described previously (Fryatt et al*.*, 2009). Briefly, the tissue sections were incubated sequentially in the following solutions: (1) 0.2% Hydrogen peroxide in methanol for 15 min; (2) 10% swine serum and 0.5% Triton X-100 in PBS for 30 min at room temperature; (3) Anti-VGSC α-subunit antibodies, as described in Table 2, overnight at 4 °C; (5) Swine anti-rabbit biotin conjugated IgG, diluted with 10% swine serum in PBS for 35 min at room temperature (DAKO, Ely, UK); (6) Streptavidin–horseradish peroxidase (HRP) conjugate for 30 min at room temperature (HRP prediluted kit, DAKO); (7) 3, 3′diaminobenzidine solution (DAB substrate kit, DAKO) for 4 min at room temperature; (8) 70% IMS for 5 min followed by 100% IMS for 5 min and 100% xylene twice for 5 min. Between steps 1 and 8 the tissue sections were thoroughly washed with PBS. Sections were mounted using DPX (RA Lamb, Eastbourne, UK).

The slides were examined using a light microscope (PriorLux, Prior Scientific Instruments Ltd, Cambridge, UK) equipped with a digital camera (Moticam 2300, GT Vision, Haverhill, Suffolk, UK) and images were captured using Motic Images Plus 2.0. ImageJ (National Institute of Mental Health, Maryland, USA) was used to measure the staining intensity using the mean grey value of the neurons from the images. For each analysis, sections that were torn, folded or partly washed off the slides were rejected, as well as sections that did not contain SGN. This yielded between 15 and 25 sections for each analysis of cell body staining in each antibody condition. The mean grey value scale ranged from: 0 = black; 255 = white. Unless otherwise stated, this scale is used throughout the results and discussion sections. Additionally, ImageJ was used to count the number of SGN cell bodies within the analysed sections. Unless stated, all micrographs shown in the results are taken from the basal third of the cochlea, position matched between the unexposed and noise exposed cochleae. Immunostaining for all VGSC isoforms was found to be similar between the cochlear apex, middle and base regions in both control and noise exposed cochlea, indicating no obvious tonotopic gradient in the rat SGN (data not shown). Histology sections showing the location of the SGN and their processes within the cochlear sections is provided in the supplementary material.

### Statistical analysis

Statistical analysis was carried out on data using both Microsoft Office Excel® 2007 spreadsheet and STATA IC Version 11.1 (StataCorp, Texas). For P1 amplitude and latency analysis, linear or polynomial lines of best fit were chosen using optimal R^2^ values, which were typically better than 0.9. Comparison of P1 latency between the first and second or first and final ABR measurements, as well as immunohistochemistry staining intensity measurements, were performed using the two-tailed Student's *t*-test.

Changes in ABR thresholds and P1 wave amplitudes over time were analysed using repeated measures ANOVA. *Post hoc* testing was carried out using paired t-tests and Bonferroni corrected for multiple comparison. These were one-tailed as noise exposure was specifically expected to result in decreased evoked auditory response. Results are expressed as mean ± SEM and the significance level was set at p < 0.05 in all experiments.

## Figures and Tables

**Supplementary Fig. 1:**
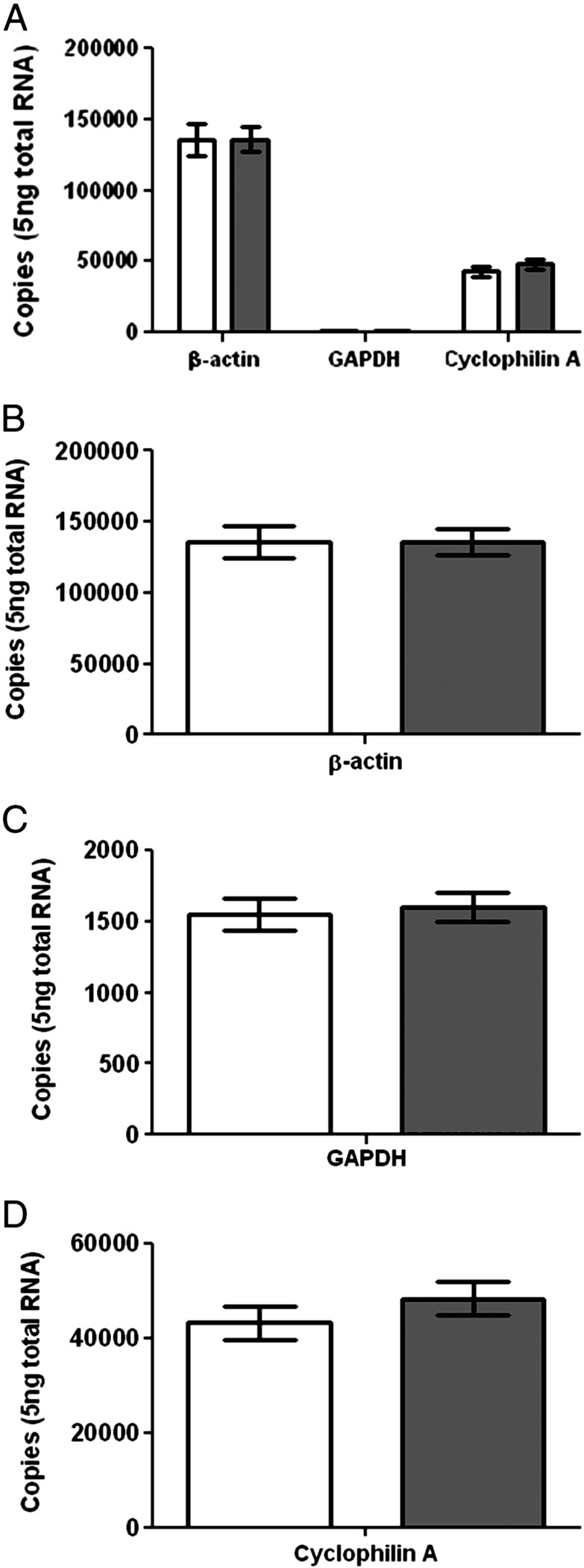
Housekeeping gene mRNA expression in control and noise exposed modioli. (A) Graph showing expression of all housekeeping genes tested, identifying β-actin, GAPDH and cyclophilin A expressed in both control (clear bars) and noise exposed (filled bars) animals. Expression of (B) β-actin, (C) GAPDH and (D) cyclophilin A mRNA was not significantly altered following noise exposure (n = 7 noise exposed, 6 control).

**Supplementary Fig. 2:**
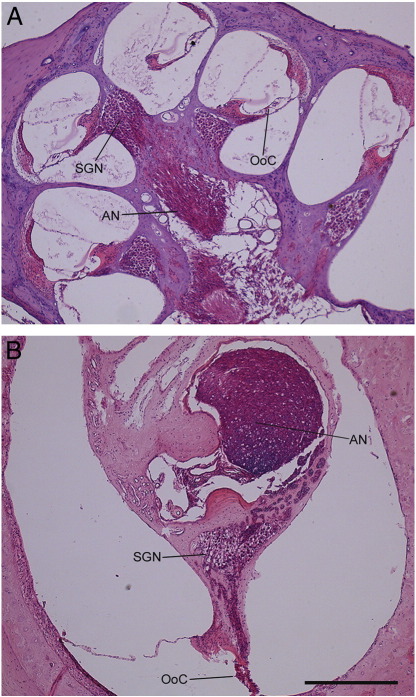
Histology of the rat cochlea. Sections of rat cochlea stained with haematoxylin and eosin showing the overall morphology. (A) Example of a cochlea sectioned in a plane parallel to the modiolus, giving sections containing the apex and base, revealing the auditory nerve (AN) within modiolus, the spiral ganglion (SGN) within Rosenthal′s canal and the organ of Corti (OoC) along the basilar membrane. (B) Example of a cochlea sectioned at approximately 90^o^ to that shown in A, showing the auditory nerve in the centre of the modiolus and the spiral ganglion forming a crescent shape. The histology sections used in this study were taken in approximately the same orientation as used to produce B. Scale bar = 500 μm.

**Table 1:** Primer and Taqman probe sequences used for qPCR.

Subunit	Oligonucleotide	Sequence 5′-3′
Na_V_1.1	Forward primer	CTGGCAGAAACCCTAACTATGGT
	Reverse primer	TCAGTCGAAACAGGGACAGAAATG
	Taqman probe	CCCAGCTGAAGGTGTC
Na_V_1.2	Forward primer	GCTCTGCTTTGTGGGAACAG
	Reverse primer	GCCTTCACACAGATGTATCCTTCT
	Taqman probe	CACTGTCCAGCATCCG
Na_V_1.3	Forward primer	CCTCTCAGACCTCGCGGACA
	Reverse primer	ACGGCAGGTCCATCTGGATT
	Taqman probe	CTCCGGCCCTCTTAGAATCCCCAAACC
Na_V_1.4	Forward primer	AATGCTAGAACTCAGCACACGGG
	Reverse primer	CTTGCTTTCAGGCAATGAGAAGAC
	Taqman probe	AAGCCAAGCCTGGTGAGCCTTCTCACT
Na_V_1.5	Forward primer	GGCTGAGGGAAGAGAGGGC
	Reverse primer	GGGAGCCTGGGAAATGGAG
	Taqman probe	AGCTGCCCACAGCTGGACACAGTTCAG
Na_V_1.6	Forward primer	CGTGATGATCCTGACAGTGTTCTG
	Reverse primer	TGAAGAGCTGCAGGCCAAT
	Taqman probe	CAGGGCGAAAACACTC
Na_V_1.7	Forward primer	TGACAGCCTGTGAAGGTTGACTC
	Reverse primer	GCCATGTGTAAATGCTGCCC
	Taqman probe	AGGCAGCACAGCCATTAGCTCTGATCC
Na_V_1.8	Forward primer	CCTGTCCATTGGGAGTCTGC
	Reverse primer	GAAGAGGAGGAGGCCGATGT
	Taqman probe	CTGCTCTTCGCCCTCATGATGTCCCT
Na_V_1.9	Forward primer	AAGCCTTTGTGTTCGACCTGG
	Reverse primer	ACATCTTTGGGCTGGTCGG
	Taqman probe	TCACAAGCCAGGTCTTTGACGTCATCA
GAPDH	Forward primer	GAACATCATCCCTGCATCCA
	Reverse primer	CCAGTGAGCTTCCCGTTCA
	Taqman probe	CTTGCCCACAGCCTTGGCAGC
Cyclophilin A	Forward primer	TATCTGCACTGCCAAGACTGA
	Reverse primer	CCACAATGCTCATGCCTTCTTTCA
	Taqman probe	CCAAAGACCACATGCTTGCCATCCA
β-actin	Forward primer	GAGCTATGAGCTGCCTGAC
	Reverse primer	AGTTTCATGGATGCCACAGGA
	Taqman probe	CATCACTATCGGCAATGAGCGGTTCC

**Table 2:** Immunohistochemistry antibodies.

Antibody	Host	Dilution	Supplier	Product code
Anti-Na_V_1.1	Rabbit	1:200	Alomone labs	ASC-001
Anti-Na_V_1.6	Rabbit	1:200	Alomone labs	ASC-009
Anti-Na_V_1.7	Rabbit	1:200	Alomone labs	ASC-008
Anti-rabbit/biotin	Swine	1:350	DAKO	E0353

Details of the antibodies used in the study. Entries with/indicates a conjugation, *i.e.* anti-rabbit/biotin means anti-rabbit conjugated with biotin. Antibodies diluted in 10% swine serum PBS for non-fluorescence immunohistochemistry.
